# Evaluation of Chagas TESA VirClia assay for chronic *Trypanosoma cruzi* infection diagnosis and post-treatment follow-up

**DOI:** 10.1128/jcm.01739-25

**Published:** 2026-06-12

**Authors:** Aroa Silgado, Alejandro Mediavilla, Patricia Martínez-Vallejo, Pau Bosch-Nicolau, Carles Rubio-Maturana, Lidia Goterris, Inés Oliveira-Souto, Núria Serre-Delcor, Joan Joseph-Munné, Israel Molina, Elena Sulleiro

**Affiliations:** 1 Department of Microbiology, Vall d’Hebron University Hospital, Universitat Autònoma de Barcelona, PROSICS Barcelona 16719, Barcelona, Spain; 2 Centro de Investigación Biomédica en Red de Enfermedades Infecciosas (CIBERINFEC), Instituto de Salud Carlos III 38176, Madrid, Spain; 3 Department of Infectious Diseases, Vall d’Hebron University Hospital, Universitat Autònoma de Barcelona, PROSICS Barcelona 16719, Barcelona, Spain; Mayo Clinic Minnesota, Rochester, Minnesota, USA

**Keywords:** chronic Chagas disease, serological diagnostics, *Trypanosoma cruzi*, CLIA assay, TESA antigen

## Abstract

Diagnosis of chronic *Trypanosoma cruzi* infection relies on the concordant results of two serological tests based on different antigens and/or principles. Currently, the only accepted cure criterion is the switch from positive to negative serology, but anti-*T*. *cruzi* IgG may persist for years, even after treatment. Recently, a novel chemiluminescence immunoassay based on native *T. cruzi* excreted-secreted antigens (TESA)—Chagas TESA VirClia—was developed. This study assessed the performance of this assay for the detection of anti-*T*. *cruzi* antibodies for the diagnosis and post-treatment follow-up of chronic *T. cruzi* infection. A retrospective diagnostic accuracy study was conducted using 227 serum samples, including samples from individuals tested for chronic *T. cruzi* infection and from individuals with other parasitic or viral infections. *T. cruzi* infection status was established using two reference serological assays based on recombinant and native antigens. All samples were analyzed by TESA-based assay and compared with the reference tests. Concordance between the TESA-based assay and the reference tests was 93.8%, with a sensitivity and specificity of 92% and 100%, respectively. Limited cross-reactivity was observed in samples positive for *Leishmania* spp. antibodies, while no cross-reactivity was observed in viral infections. A decrease in antibody index over time was observed more frequently in treated patients when tested by the TESA-based assay. These findings support the use of the Chagas TESA VirClia assay as a reliable confirmatory tool within the diagnostic algorithm for chronic *T. cruzi* infection. Moreover, this assay was the only test showing a consistent post-treatment decline in antibody index over time.

**IMPORTANCE:**

The diagnosis and monitoring of chronic *Trypanosoma cruzi* infection remain challenging because no single serological test achieves 100% sensitivity and specificity, and the antibodies may persist for many years even after treatment. This study highlights the value of a novel serological assay based on native antigens excreted by the parasite, which more effectively reflects changes in antibody levels over time. Its high diagnostic accuracy and greater ability to detect declining antibody values in treated patients support its use as a reliable diagnostic tool as well as a promising method for serological follow-up.

## INTRODUCTION

Chagas disease (CD), a parasitic infection caused by the parasite *Trypanosoma cruzi*, is endemic in continental Latin American countries and is estimated to affect 6–7 million people worldwide (1). Due to migratory movements out of the endemic area, people with *T. cruzi* infection have been increasingly detected beyond Latin America. Specifically, Spain has the largest population of imported cases of *T. cruzi* infection, second only to the United States (2).

Clinical infection with *T. cruzi* occurs in two phases: acute and chronic. The acute phase is usually asymptomatic and is followed by a chronic phase if untreated. While most remain asymptomatic for life, 30%–50% of chronically infected individuals eventually develop cardiac and/or digestive complications (3). The diagnosis in this phase is based on the detection of anti-*T*. *cruzi* IgG antibodies. Serological techniques use whole-lysate antigens (conventional techniques) or recombinant/synthetic peptides (non-conventional techniques). Indirect immunofluorescence, indirect hemagglutination, and enzyme-linked immunosorbent assays (ELISA) are the most common conventional tests used. On the other hand, non-conventional tests include ELISA or chemiluminescence immunoassay (CLIA). Although several immunological tests and methods based on different antigens and/or technological platforms are available, there is no serological reference test for the diagnosis of chronic *T. cruzi* infection. Thus, the World Health Organization guidelines recommend performing at least two serological tests in parallel with different principles and/or antigens (4). Moreover, it should be noted that the use of two different serological tests may lead to discordant results, and a third technique or additional samples are required (5).

The *T. cruzi* trypomastigote excreted-secreted antigen (TESA) is widely used as a confirmatory assay for chronic *T. cruzi* infection in immunoblot or ELISA formats (6, 7) but is currently limited to reference centers for in-house testing (8).

New-generation techniques have significantly improved the accuracy of chronic *T. cruzi* infection analytical diagnosis by incorporating the use of a large mixture of recombinant antigens and different detection systems (9). A novel test using the TESA for the first time in a CLIA assay (Chagas TESA VirClia) has been developed. This technique is presented as a fully automated assay in a monotest format, allowing the test to be performed on demand and the reagents and quality controls to be ready for use.

On the other hand, serology also remains the method of choice for determining cure in chronically infected patients. Currently, only two drugs, benznidazole and nifurtimox, are approved for the treatment of CD (10). The general consensus is that treatment success is confirmed by the switch from a positive to a negative serological state (11). Nevertheless, anti-*T*. *cruzi* IgG antibodies can be detected over several years using current serological tests, even after treatment (12). This underlines the need for a serological tool to timely monitor the response to treatment of CD.

The aim of this study was to assess the performance of the Chagas TESA VirClia assay for the diagnosis and follow-up of chronic *T. cruzi* infection.

## MATERIAL AND METHODS

### Study design and serum samples

**Fig 1:**
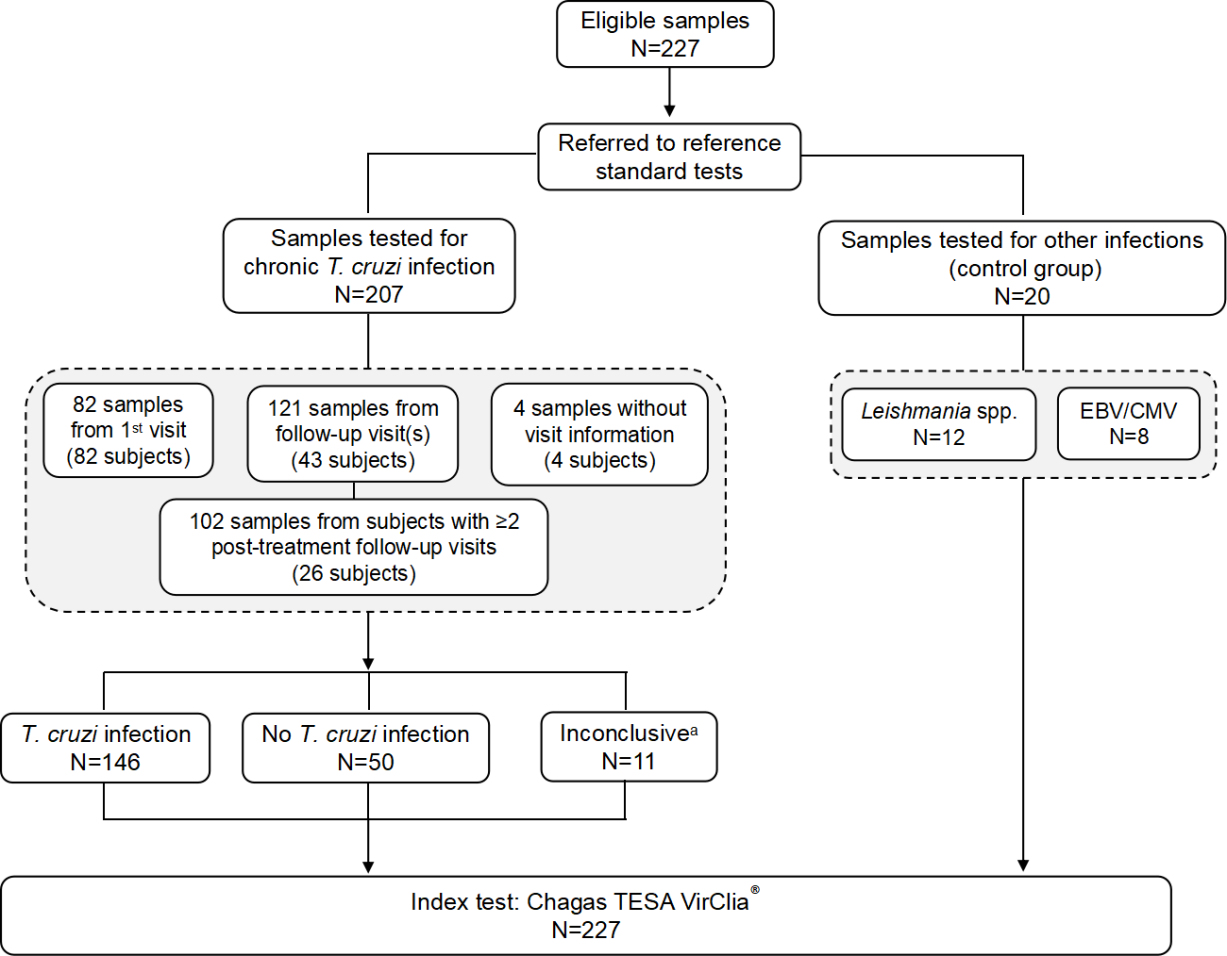
Flowchart description of the study samples according to the Standards for Reporting of Diagnostic Accuracy Studies (STARD) guidelines. ^a^Inconclusive: discrepant results between reference standard tests. EBV, Epstein-Barr Virus; CMV, Cytomegalovirus; TESA, *T. cruzi* trypomastigote excreted-secreted antigens.

A retrospective diagnostic accuracy study was performed using 227 stored serum samples collected between 2017 and 2023 at the Microbiology Department of Vall d’Hebron University Hospital (HUVH) (Fig. 1).

Samples were selected according to their predefined infection status and classified into two groups: (i) 207 samples from individuals screened for or monitored due to chronic *T. cruzi* infection, and (ii) 20 samples from patients diagnosed with infections other than *T. cruzi*, including *Leishmania* spp. (visceral form, *n* = 12) or Epstein-Barr/Cytomegalovirus (EBV/CMV) infection (*n* = 8).

Out of the total samples tested for chronic *T. cruzi* infection (*n* = 207), 82 samples corresponded to the initial diagnostic visit, while the remaining 121 samples were collected during post-treatment follow-up visit(s). Information regarding the type of visit could not be determined in four cases.

Among the follow-up samples, 102 samples corresponded to 26 adult treated individuals who had two or more post-treatment follow-up visits (at least 1 year after treatment and 1 year apart between visits). These individuals underwent annual follow-up in the Infectious Diseases Department of HUVH.

### Testing procedures

*T. cruzi* infection status was established based on the consensus results of two reference serological assays for the detection of anti-*T*. *cruzi* antibodies: serum samples were tested for recombinant antigen using an electrochemiluminescence immunoassay (E-CLIA) (Elecsys Chagas, Roche Diagnostics, Mannheim, Germany). Those with a positive result were subsequently analyzed for a whole-lysate antigen using a commercial ELISA (Ortho *T. cruzi* ELISA, Johnson & Johnson, High Wycombe, United Kingdom). Samples with discordant results between these assays were considered inconclusive, as definitive classification was not possible in the absence of a third confirmatory test.

All serum samples were initially stored at −20°C for 1–2 months before transfer to −80°C for long-term storage.

Serum samples were analyzed retrospectively using the Chagas TESA VirClia assay (Vircell S.L., Granada, Spain), according to the manufacturer’s instructions, with the results of the reference serological tests blinded to the operator.

For the Elecsys Chagas assay, samples with a cutoff (CO) index (COI) value of ≥1.0 were considered reactive (i.e., positive for antibodies to *T. cruzi*), and those with a COI value of <1.0 were considered non-reactive (i.e., negative for antibodies to *T. cruzi*).

In our laboratory, ELISA results are classified as reactive or non-reactive according to the manufacturer’s cutoff, and an internal analytical gray zone is defined as values within ±10% of the CO. Thus, for the Ortho *T. cruzi* ELISA assay, which does not define a gray zone in the package insert, samples were interpreted as reactive if values were higher than or equal to the CO and non-reactive if values were lower than the CO, while those within ±10% of the CO were considered in the gray zone.

For the Chagas TESA VirClia assay, samples were considered reactive if signal/CO (S/CO) values were >1.1, inconclusive if S/CO values were between 0.9 and 1.1 (gray zone), and non-reactive if S/CO values were <0.9.

### Statistical analysis

Sensitivity and specificity values were calculated to determine the diagnostic accuracy of the Chagas TESA VirClia assay against the reference diagnostic algorithm. The Cohen Kappa coefficient was used to analyze the level of agreement between tests and interpreted as follows: slight (0.00–0.20), fair (0.21–0.40), moderate (0.41–0.60), substantial (0.61–0.80), and almost perfect agreement (0.81–1.00).

Samples with discordant results between reference assays and samples with “gray zone” results by Chagas TESA VirClia assay were excluded from sensitivity and specificity calculations. However, these samples were included in agreement analyses performed separately with each reference assay.

Qualitative variables were expressed as absolute frequencies and percentages, and quantitative variables as the median and interquartile range (IQR). Continuous variables were compared using the *t*-test or Mann-Whitney U test when appropriate. Cochran’s Q test was used to analyze the differences in the proportion of patients exhibiting a decline in the antibody index values over time between serological techniques. Significance was determined at a *P*-value <0.05 for all cases.

To analyze the longitudinal dynamics of antibody levels for each serological assay, a linear mixed-effects model was applied. Model parameters were estimated using restricted maximum likelihood (REML).

Statistical analyses were done using SPSS Statistics v22.0 (IBMP Corp., Armonk, NY) and R Statistical Software (v4.5.0; R Core Team 2025).

## RESULTS

### Diagnostic accuracy of Chagas TESA VirClia assay compared with conventional serological testing

According to the reference algorithm, 146/207 (70.5%) serum samples were positive for anti-*T*. *cruzi* antibodies, 50/207 (24.2%) were negative, and 11/207 (5.3%) samples were classified as inconclusive (i.e. discrepant results between the reference serological assays). Of these 11 samples, all tested positive by the Elecsys Chagas assay; however, eight samples were negative, and three had a result in the gray zone by the Ortho *T. cruzi* ELISA. All 11 samples with an inconclusive result were negative by the Chagas TESA VirClia assay. Additionally, 3/207 samples (1.4%) returned a “gray zone’’ result by Chagas TESA VirClia (all positive by both Elecsys and Ortho assays).

A total of 14 samples (11 with an inconclusive result by the reference algorithm and 3 with a gray zone result by Chagas TESA VirClia) were excluded from the diagnostic accuracy analysis.

Overall concordance for the Chagas TESA VirClia with the reference algorithm was 93.8%. The analytical performance of the Chagas TESA VirClia assay is shown in Table 1.

**TABLE 1:** Analytical performance of Chagas TESA VirClia assay against the reference tests for the diagnosis of chronic *T. cruzi* infection (*N* = 193)

	*T. cruzi* infection status		Sensitivity % (95% CI^*a*^)	Specificity % (95% CI)	Kappa value (95% CI)
Chagas TESA^*b*^ VirClia	Infection	No infection			
Positive	131	0	91.6 (85.5–95.4)	100 (91.1–99.8)	0.850 (0.77–0.93)
Negative	12	50			

^*a*^ CI, confidence interval.

^*b*^ TESA, *T. cruzi* trypomastigote excreted-secreted antigens.

Twelve out of 193 samples (6.2%) reported a discrepant result between Chagas TESA VirClia and the reference tests. The antibody index values obtained by both Elecsys Chagas and Ortho *T. cruzi* ELISA indicate that these 12 samples have low values of IgG anti-*T*. *cruzi* (Table 2).

**TABLE 2:** Antibody index values obtained in samples with discrepant results (negative by Chagas TESA VirClia and positive by both Elecsys Chagas and Ortho *T. cruzi* ELISA; *N* = 12) compared to samples positive by the three serological techniques

Assay	Positive concordant samples^*a*^ (*n* = 131)	Discrepant samples (*N* = 12)	*P*-value
Elecsys Chagas	237.5 (196.8–282.3)	48.05 (19.29–81.98)	<0.001
Ortho *T. cruzi* ELISA	7.21 (6.18–8.56)	1.69 (1.48–1.99)	<0.001
Chagas TESA VirClia	3.02 (2.06–4.25)	0.38 (0.36–0.48)	<0.001

^*a*^ Results are expressed as median and interquartile range (Q1–Q3).

The performance of the Chagas TESA VirClia technique, in terms of agreement, was assessed in comparison with Elecsys Chagas and Ortho *T. cruzi* ELISA methods, stratified by the type of visit (Table 3). For samples corresponding to the diagnostic visit, Chagas TESA VirClia demonstrated a concordance of 97.6% with the Elecsys Chagas assay, while achieving a concordance of 100% when compared to Ortho *T. cruzi* ELISA. However, when samples from follow-up visits were analyzed, the concordance of Chagas TESA VirClia dropped to 83.9% and 90.5% when compared to Elecsys Chagas and Ortho *T. cruzi* ELISA assays, respectively.

**TABLE 3:** Concordance of Chagas TESA VirClia assay results with reference tests results for the detection of anti-*T*. *cruzi* antibodies according to the type of visit (1st visit or follow-up visits)

	Chagas TESA VirClia						
	Screening visit (*n* = 82)*^a^*			Follow-up visit (*n* = 121)^*a*^			
	N	Positive N (%)	Negative N (%)	N	Positive N (%)	Negative N (%)	Gray zone N (%)
Elecsys Chagas							
Positive	32	**30 (93.8)**	2 (6.2)	121	**99 (81.8)**	19 (15.7)	3 (2.5)
Negative	50	0	**50 (100)**	0	0	0	
Ortho *T. cruzi* ELISA							
Positive	30	**30 (100)**	0	113	**99 (87.6)**	11 (9.7)	3 (2.7)
Negative	51	0	**51 (100)**	6	0	**6 (100)**	
Gray zone	1		1 (100)	2		2 (100)	

^*a*^ Bolded numbers indicate concordance.

### Cross-reactivity analysis

**Fig 2:**
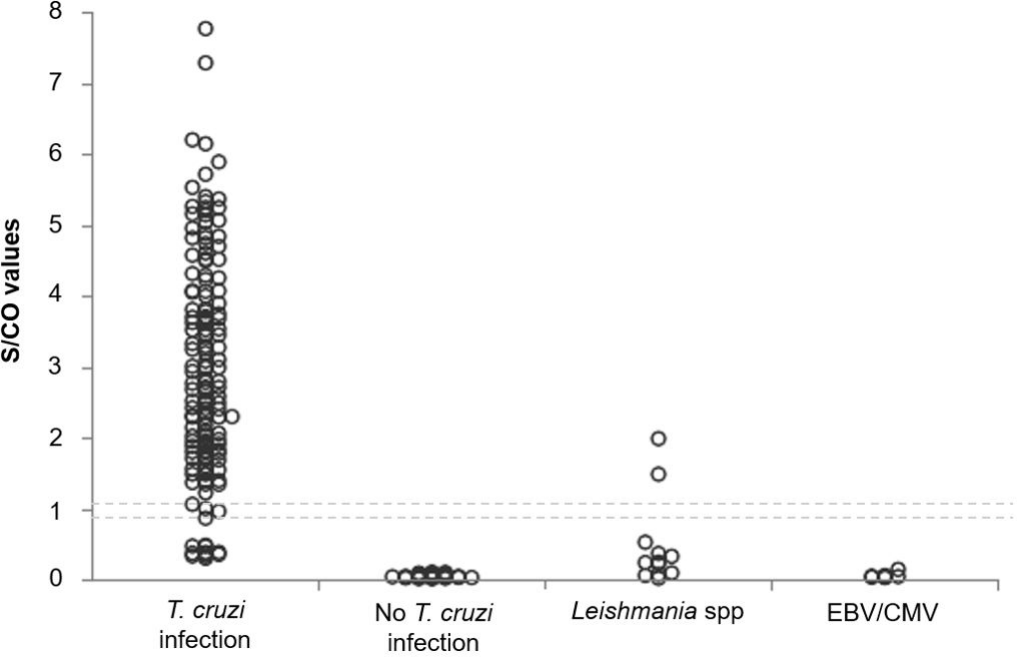
Chagas TESA VirClia S/CO values according to infection status. Dashed lines set limits of the gray zone (range of 1.1–0.9 S/CO values). EBV, Epstein-Barr virus; CMV, Cytomegalovirus.

A cross-reactivity to Chagas TESA VirClia assay occurred in 2/12 (16.7%) *Leishmania* spp. positive sera. All eight (100%) samples from patients with EBV/CMV infection returned negative results by Chagas TESA VirClia assay (Fig. 2).

### Monitoring of serum antibody index over time in treated patients

Twenty-six treated patients had ≥2 post-treatment follow-up visits, with a median age of 48 years (range: 39–64), and a majority of females (73%). All participants (26/26; 100%) received benznidazole as treatment. Most participants (22/26; 85%) completed the treatment regimen.

No statistically significant association was observed between treatment completion or follow-up duration, including participants with extended follow-up periods (e.g., 9 years or more), and the probability of a decline in antibody levels across any of the serological assays.

**Fig 3:**
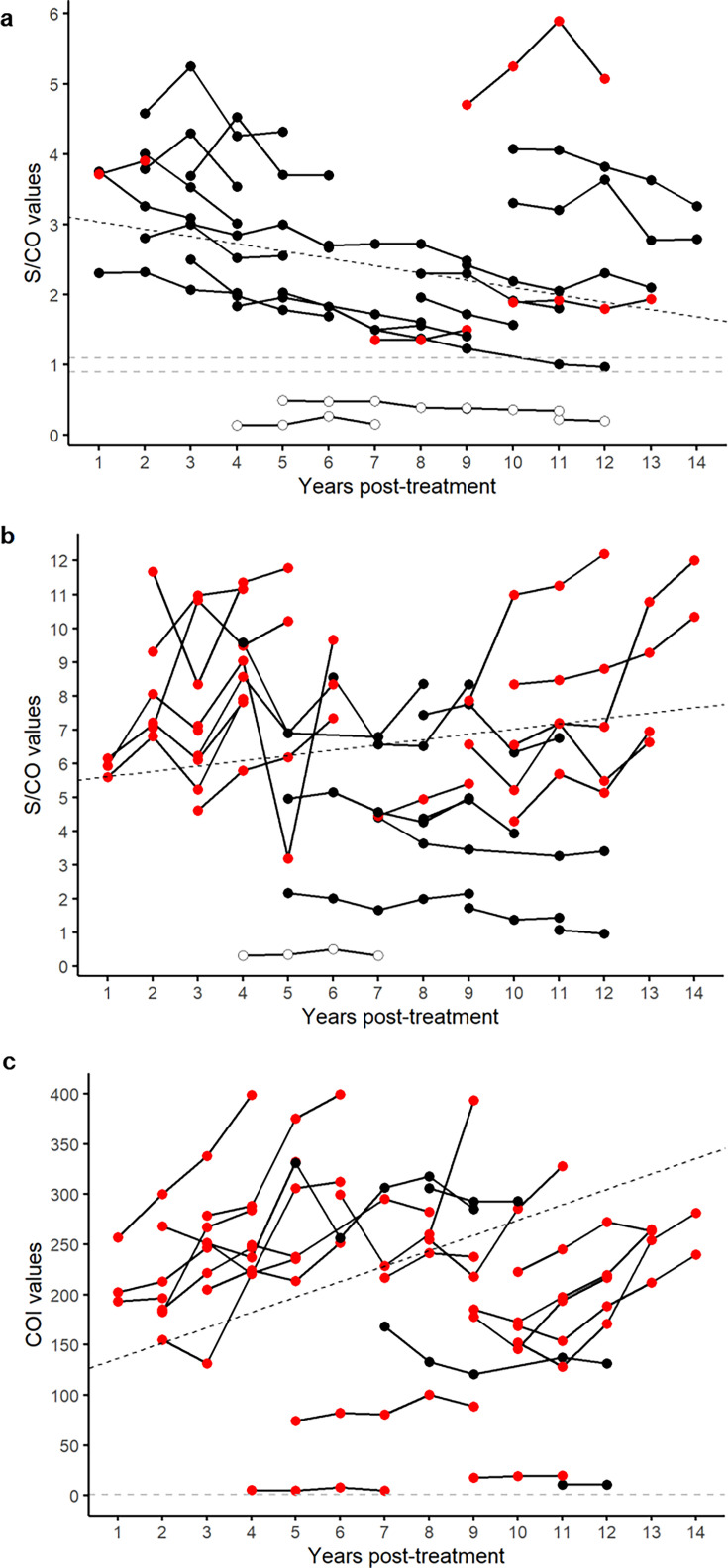
Dynamics of anti-*T. cruzi* antibody index over time in treated patients. (**a**) S/CO values in samples tested by the Chagas TESA VirClia. (**b**) S/CO values in samples tested by the Ortho *T. cruzi* ELISA system, and (**c**) COI values in samples tested by the Elecsys Chagas assay. Each line represents one individual (*n* = 26). Patients with a decrease in antibody index over follow-up visits are represented with black circles; patients with an increase in antibody index with red circles. Unfilled black circles represent those samples with a negative result by the corresponding serological assay. Gray-dashed lines set limits of the gray zone (range of 1.1–0.9) for the Chagas TESA VirClia assay or the cutoff value (≥1.0) for the Elecsys Chagas assay. The dashed-black line represents the estimated global mean trajectory derived from the linear mixed-effects model for each assay. S/CO, sample/cutoff value; COI, sample cutoff index.

A decline in anti-*T. cruzi* antibody index over time was observed in a higher proportion of patients when samples were analyzed using the Chagas TESA VirClia assay, as compared to the Ortho *T. cruzi* ELISA or Elecsys Chagas assays: 22/26 (84.6%), 9/26 (34.6%), and 4/26 (15.4%) patients, respectively (*P*-value<0.001) (Fig. 3). Moreover, the Chagas TESA VirClia assay was the only unique assay that showed an overall decrease in antibody titer over time (Fig. 3).

## DISCUSSION

The present study investigated the Chagas TESA VirClia assay for the diagnosis and post-treatment follow-up of chronic *T. cruzi* infection. The findings obtained in this study, in line with those reported by Garcia-Bermejo et al. (13), demonstrated a sensitivity and specificity of 92% and 100% for the Chagas TESA VirClia and a performance comparable with the reference algorithm (Kappa value >0.80). The analysis of samples with discrepant results between the Chagas TESA VirClia assay and the reference tests revealed significantly lower anti-*T. cruzi* antibody titers across all three assays compared to those concordant positive samples, with higher antibody titers. This finding suggests that the Chagas TESA VirClia assay may exhibit reduced sensitivity in detecting samples with low-titer antibodies.

According to the latest guidelines (4, 14), the recommended diagnostic algorithm for patients with suspected chronic *T. cruzi* infection consists of the use of a high sensitivity assay initially, followed by confirmation with a high specificity assay. In this regard, the Chagas TESA VirClia assay achieved a high concordance with Elecsys Chagas and Ortho *T. cruzi* ELISA assays for samples corresponding to the diagnostic visit (98% and 100%, respectively). It is well known that recombinant antigen-based tests (such as the Elecsys Chagas) exhibit higher sensitivity in contrast to native antigen-based tests as a first-line screening test for the diagnosis of chronic *T. cruzi* infection (15, 16).

On the other hand, the Chagas TESA VirClia assay performs optimally for use as a confirmatory test in the serological diagnostic algorithm of chronic *T. cruzi* infection, due to the high specificity obtained and also the perfect concordance with the confirmatory assay used in the present work (Ortho *T. cruzi* ELISA). The Ortho *T. cruzi* ELISA assay, previously a standard for the Food and Drug Administration (FDA)-approved donor screening (17), is being discontinued following QuidelOrtho’s announced phased exit from the U.S. donor testing market by the end of 2025, alongside the other assays. This situation highlights the potential of Chagas TESA VirClia as a native antigen-based alternative and a suitable replacement for Ortho ELISA in confirmatory testing.

Although ELISA-based techniques have been widely used for the diagnosis of chronic *T. cruzi* infection (17), new-generation methods, such as CLIA, are increasingly becoming popular for detecting anti-*T. cruzi* antibodies. Immunoassays based on CLIA are well-established products for *in vitro* diagnosis, running on high-throughput instruments, allowing for increased sensitivity, automation, and turnaround time of assays (18). Specifically, Chagas TESA VirClia is presented in a monotest format that allows performing the assay on demand and uses native TESA antigen for the first time in a CLIA-based immunoassay.

The discrepancies found between Chagas TESA VirClia and Elecsys Chagas and Ortho *T. cruzi* ELISA assays were mainly observed in those samples collected during post-treatment follow-up visits. Cases with discordant results between techniques or with index values close to the cutoff may be in a phase of antibody decline that could lead to seroreversion in the long term (5). In this sense, a major advantage of the Chagas TESA VirClia assay lies in its superior ability to detect a decrease in anti-*T. cruzi* antibody levels over time. Among the 26 treated patients with serial post-treatment follow-up samples, the majority exhibited a significant decline in antibody index values over time when using the Chagas TESA VirClia assay, whereas less than 35% of treated patients exhibited similar trends using the Ortho *T. cruzi* ELISA and Elecsys Chagas assays. These findings are consistent with prior research suggesting that the TESA-based assay identifies a higher proportion of treated patients as negative compared to recombinant antigen-based ELISA (19). This feature is particularly relevant in the context of CD, where the persistence of antibodies for years after treatment can complicate the determination of cure. Thus, enhanced detection of declines in antibody levels could enable more timely and accurate assessments of therapeutic outcomes.

Our analysis revealed no significant correlation between treatment completion or duration of follow-up and the probability of detecting a decline in antibody levels over time using any of the serological methods. The lack of association with longer follow-up is particularly noteworthy, as it underscores the slow and heterogeneous dynamics of serological evolution after treatment in chronic CD (20) and emphasizes the limitations of using conventional serology alone to define cure or treatment success.

Cross-reactivity on TESA-based assays has previously been reported for subjects with *Leishmania* spp. infection (6, 13). Although two cross-reactivity cases with *Leishmania*-positive sera were detected, the high specificity exhibited by the Chagas TESA VirClia assay should not result in a significant compromise to its utility in the diagnostic algorithm for chronic *T. cruzi* infection. Previous studies have described the fact that the use of recombinant antigen as a first screening test has helped overcome the problem of cross-reactions described more frequently in conventional tests (21).

Our study, however, has limitations due to its retrospective nature. First, the absence of a third reference method in our study prevented definitive classification of discordant cases. In routine clinical practice, serodiscordance in chronic *T. cruzi* infection diagnosis represents a significant challenge and often requires additional confirmatory testing (4, 5). Additionally, the relatively small number of negative samples may affect the precision of specificity estimates, and larger studies including broader negative panels would be valuable. Second, for patients with several post-treatment follow-up visits, the complete follow-up was not available. Likewise, in order to determine accurately the dynamics of the antibody index over time, it would have been appropriate to have the pre-treatment sample available. Moreover, the lack of clinical variables hinders the interpretation of the observed antibody dynamics, as it is unclear how these serological trends translate into disease progression or parasitological cure. Furthermore, no untreated comparison group was included to determine if the decline in antibody index over time detected by the Chagas TESA VirClia would occur regardless of treatment. These limitations, along with the relatively small sample size for the post-treatment group, prevent firm conclusions and limit the generalizability of our findings. Taken together, further studies should be pursued with a well-defined patient cohort with pre-treatment baseline samples, longer follow-up, and control groups to have better assess antibody dynamics over time.

To conclude, the Chagas TESA VirClia assay demonstrates robust diagnostic accuracy, high specificity, and reliable performance for detecting declines in antibody levels over time after treatment. These findings support the incorporation of the Chagas TESA VirClia assay into routine clinical workflows as a confirmatory test in the serological diagnostic algorithm for chronic *T. cruzi* infection. Moreover, Chagas TESA VirClia was the only assay demonstrating a consistent post-treatment decline in antibody index value over time, although further studies are required to determine its role in monitoring treatment response.

## Data Availability

The original contributions presented in the study are included in the article. Further inquiries should be directed to the corresponding author.
